# Protein intake, malnutrition, and its association with bone health after a hip fracture: A 3-month prospective study

**DOI:** 10.3934/publichealth.2025048

**Published:** 2025-09-26

**Authors:** Inge Groenendijk, Hugo H Wijnen, Diana G Taekema, Lisette CPGM de Groot

**Affiliations:** 1 Division of Human Nutrition and Health, Wageningen University & Research, Wageningen, The Netherlands; 2 Department of Geriatrics, Rijnstate Hospital, Arnhem, The Netherlands

**Keywords:** protein, nutritional status, bone, bone mineral density, hip fracture patients

## Abstract

**Background:**

In this study, we describe the change in protein intake, nutritional status, bone markers, and bone mineral density (BMD) in older patients recovering from a hip fracture, from post-surgery till 3 months. Additionally, we explore the association between protein intake with bone markers, quantitative ultrasound (QUS) and BMD, and nutritional status with QUS and BMD.

**Methods:**

A 3-month prospective study in 96 adults aged ≥70 years with an acute hip fracture was conducted. Assessments after surgery and 3 months included protein intake (questionnaire), nutritional status [Mini Nutritional Assessment Short Form (MNA-SF)], procollagen type I N-terminal propeptide (PINP), C-terminal telopeptide of type I collagen (CTX), insulin-like growth factor 1 (IGF-1), parathyroid hormone (PTH) levels, QUS parameters, and BMD (dual-energy X-ray absorptiometry). Associations were assessed by adjusted linear mixed models.

**Results:**

At baseline, half of the patients (mean age 84 years, 63% females) had a low protein intake (<0.8 g/kg/d), which did not change over time. The patients had significant weight loss (median 3.6 kg) and the prevalence of (being at risk of) malnutrition increased from 20% to 64%. The PINP and IGF-1 levels increased over time, the CTX level remained stable, and the PTH level decreased. The protein intake was only associated with a QUS parameter in females (estimate 0.123, 95% *CI* 0.022–0.223). A higher pre-fracture MNA-SF status was associated with higher BMD in the total body (estimate 0.048, 95% *CI* 0.015–0.080), spine (estimate 0.085, 95% *CI* 0.025–0.144), total hip (estimate 0.055, 95% *CI* 0.018–0.093), and trochanter (estimate 0.057, 95% *CI* 0.018–0.096). IGF-1 was associated with PINP (estimate 1.215, 95% *CI* 0.363–2.066).

**Conclusions:**

A good nutritional status is associated with higher BMD in older hip fracture patients. The role of protein for bone health in these patients remains unclear. After a hip fracture, there is an increase in PINP.

## 1. Introduction

Hip fractures have a major impact on the health status and quality of life of patients [1]. Only half of the older hip fracture patients regain their pre-fracture functional level [2], and 24% of the patients die within the following year [3]. In addition, an increased loss of bone mineral density (BMD) can be observed after the sustained hip fracture [4],[5].

Since a higher dietary protein intake may reduce the hip fracture risk, maintain BMD and prevent BMD loss in older adults [6],[7], this nutrient may play a beneficial role in the recovery of hip fractures. Previously, it was found that the mean protein intake was insufficient in older hip fracture patients in geriatric rehabilitation wards (<0.8 in 46% and <1.2 g/kg/day in 92%) [8]. Similar findings have also been observed during hospital stays [9]–[11]. A low protein intake reduces the insulin-like growth factor 1 (IGF-1) levels and may increase the parathyroid hormone (PTH) levels, which leads to a reduction in bone formation and a stimulation of bone resorption, respectively [12]–[14]. Therefore, a high protein intake may relate to a higher BMD.

BMD is used as an indicator of bone health; however, a dual-energy X-ray absorptiometry (DXA) scan is a costly and non-portable method, which is difficult to perform on patients with a recent hip fracture. An alternative method to assess bone health is measuring bone turnover markers, which are produced during bone remodeling. Bone undergoes continuous remodeling through bone resorption by osteoclasts, followed by bone formation by osteoblasts [15]. While bone remodeling is in balance in healthy young adults, an increased bone turnover rate is present in older adults, which results in a net bone loss [15],[16]. Additionally, an increased bone turnover is associated with a higher fracture risk [15],[16]. However, an increased bone metabolism is needed to reestablish bone structure following a fracture. A previous study in hip fracture patients found that the procollagen type I N-terminal propeptide (PINP, bone formation marker) levels increased from baseline to 2 months, while the C-terminal telopeptide of type I collagen (CTX, bone resorption marker) levels did not change from baseline to 2 months [17]. However, the baseline measurement in that study was performed 2 weeks post-fracture, meaning that the levels were probably already elevated. A study in Chinese hip fracture patients measured the PINP and CTX levels before surgery and from 30–60 days, 80–120 days, and 180–230 days after surgery [18]. The levels of both markers peaked at 30–60 days after surgery. Since the follow-up measurements were determined within broad time ranges, the exact trajectory is unknown. However, the levels of PINP and CTX just after hip surgery and how they change within 3 months remain unknown (catabolic state most pronounced [19]). Next to bone turnover markers, another non-invasive method that reflects bone health is the quantitative ultrasound (QUS) of the calcaneus. QUS parameters have been correlated with BMD and can predict the risk for a future fracture [20].

A limited number of studies investigated the effect of protein intake on bone turnover markers and QUS. Two studies in community-dwelling older adults found no differences in bone markers between high *vs*. low protein intake after 3 years of follow-up [21],[22]. However, they used serum osteocalcin and urinary N-telopeptide cross-links levels, which tend to change less than the PINP and CTX levels [16]. In addition, no turnover rate was calculated. Regarding the QUS, a study in Korean adults that consumed relatively low protein diets found that the meat protein intake was positively associated with one of the QUS parameters in older men, but not in older women [23]. To our knowledge, no studies have investigated the association between protein intake with bone turnover markers PINP and CTX, and the QUS parameters in hip fracture patients.

Low dietary intake, including low protein intake, is one of the determinants of malnutrition [24]. Malnutrition is common in older hip fracture patients and increases the risk of post-fracture complications and mortality [25]. Malnutrition may result in a lower BMD, as it can lead to deficiencies in essential nutrients that support bone health such as calcium, vitamin D, and protein [26],[27]. In addition, rapid weight loss is associated with a decrease in BMD [26],[28]. The association between nutritional status as measured with the Mini Nutritional Assessment Short Form (MNA-SF) with QUS and BMD has not been investigated in hip fracture patients. However, there are indications that a higher MNA score is associated with higher QUS parameters in older adults [29],[30].

In this study, we describe the change in protein intake, nutritional status, bone markers (IGF1, PTH, PINP, and CTX), and bone health (QUS, BMD) in older patients recovering from a hip fracture, from post-surgery till 3 months. Additionally, we explore the association between protein intake with bone markers and QUS and BMD, and nutritional status with QUS and BMD.

## 2. Materials and methods

### 2.1. Study design and population

A 3-month prospective study was conducted at Rijnstate Hospital (Arnhem, NL). A total of 96 older adults (aged 70 years or older) with an acute hip fracture were recruited. In addition, patients were eligible if they lived at home and had a pre-fracture Clinical Frailty Scale (CFS) score ≥3 and <7. The exclusion criteria are as follows: a pathological or periprosthetic fracture; current participation in scientific research that interferes with the current study; history of dementia; and no permission to request information about their medical history, medication use, and liver and kidney values.

The study was conducted according to the principles of the 1964 Declaration of Helsinki and its later amendments, and registered at ClinicalTrials.gov as NCT05039879. This study was approved by an appropriate Ethics Committee (“METC Oost-Nederland”) and a declaration confirming that the Medical Research Involving Human Subjects Act does not apply was obtained. After being fully informed, the patients gave explicit oral consent to participate in the study, which was recorded in the electronic patient record.

### 2.2. Study procedures

The measurements were performed at baseline on the traumatology ward (within 5 days after hip surgery) and after 3 months during an outpatient clinic visit to evaluate the patient's physical health and cognitive functioning.

#### 2.2.1. Protein intake and nutritional status

The dietary protein intake was recorded using a protein screener developed by hospital Gelderse Vallei (Ede, NL). This tool aims to reflect the protein intake (g/d) based on their eating habits and specifically focuses on food products which are high in protein. For this study, the patients were asked about the frequency and quantity they consumed of each food product per day or week, thereby considering their habitual intake in the past month.

The nutritional status was assessed with the MNA-SF [31]. The subjects were classified as follows: having a normal nutritional status (12–14 points); being at risk of malnutrition (8–11 points); and being malnourished (0–7 points).

#### 2.2.2. Calcaneal quantitative ultrasound

The QUS parameters of the calcaneus (heel) were measured using a portable Achilles EXPII bone ultrasonometer (GE Healthcare, USA). This device emits sound waves at a high frequency (ultrasonic). The coefficient of variation (CV) was <2.0%. The measurement was performed in a seated position, and both the right and left calcaneus were measured in duplo. From the broadband ultrasound attenuation (BUA) and speed of sound (SOS) parameters, a Stiffness Index (SI) was calculated using the following formula: SI = 0.67 * BUA + 0.28 * SOS − 420 [32]. The mean BUA, SOS, and SI were calculated as the average of the left and right calcaneus. When only one side could be assessed, the mean of one side was taken.

#### 2.2.3. Bone markers and vitamin D status

The PINP and CTX bone turnover markers were measured in serum in the hospital using a chemiluminescent immunoassay (CLIA; Liaison XL, Diasorin) [16]. For the measurement of CTX levels, the intra-assay and inter-assay CV were 2.1%–4.9% and 5.1%–8.8%, respectively. For the measurement of PINP levels, the intra-assay and inter-assay CV were 2.6%–3.0% and 4.2%–5.3%, respectively. Since the CTX levels are highly affected by circadian rhythm, blood samples were taken in the morning before 10 AM for each patient and each time point [16]. Moreover, the CTX levels are affected by diet; therefore, the patients needed to fast overnight before the blood sample collection [15]. The IGF-1 and PTH levels in the blood were measured using CLIA as well and the inter-assay CV were 4.7%–5.3% and 2.3%–7.8%, respectively. Lastly, the serum 25(OH)D level was measured using an electrochemiluminescence immunoassay (inter-assay CV was 2.8%–6.9%).

#### 2.2.4. Other measurements

Two questionnaires were used to assess the clinical frailty and activities of daily living. The CFS (version 2.0) [33],[34] is already used in daily practice to assess the overall level of fitness and frailty of older patients. This scale is graded from 1 to 9, in which level 1 represents “very fit”and level 9 represents “terminally ill”. An experienced clinician evaluated the frailty status. The activities of daily living were assessed with the Barthel Index [35],[36], which includes 10 different activities of daily living. A score between 0–20 is utilized, where 0 means fully dependent and 20 means totally independent.

The following outcomes were extracted from the patient files: socio-demographic characteristics (sex, age, smoking and drinking habits, living situation), hip fracture details (cause, location, type of surgery), length of hospital stay and discharge destination, length of rehabilitation, complications, fracture history, estimated glomerular filtration rate (eGFR), C-reactive protein (CRP) and leukocytes, comorbidities using the Charlson Comorbidity Index (CCI), and mortality. Additionally, the smoking and drinking habits and fracture history were verified with the patient. In addition, the patients were asked about their education level, which was classified as either primary, lower secondary, upper secondary, and higher education.

Medication that potentially affects bone health was recorded by the clinician at discharge, which included corticosteroids, bisphosphonates or other osteoporosis medication, hormones (i.e., estrogen, hormone replacement therapy), and vitamin D and calcium supplements.

The patient's bodyweight (kg) was measured with a calibrated sitting weighing scale at baseline and at 3 months, while their height was measured to the nearest 1 cm with a stadiometer. For patients who could not stand, the knee length was measured while either sitting or lying down with a straight ruler (SECA). The total height was calculated using formulas specifically developed for older adults [37]. Subsequently, the patient's BMI was calculated as bodyweight divided by height in meters squared.

Two measurements were solely assessed at 3 months: memory (Montreal Cognitive Assessment, MoCA [38]) and DXA (Lunar iDXA, GE Healthcare, USA). Using DXA, the BMD (g/cm^2^) of multiple sites (total body, spine, lumbar spine, total hip, femoral neck, Ward's triangle, trochanter and pelvis) and bone mineral content (BMC; g) were collected. The unfractured site was measured for the hip parameters.

### 2.3. Statistical analysis

A power calculation for the associations between protein intake and bone health was based on the rule that there should be at least 10 observations per variable [39]. The intention was to make models with a maximum of 8 variables, which led to a sample size of 80 patients. With an expected drop-out of 20%, a sample size of 96 hip fracture patients was needed.

The data are expressed as mean ± standard deviation (*SD*), *n* (%), or as a median with an interquartile range (*IQR*) for non-normally distributed data. The data were checked for normality using histograms and the Shapiro-Wilk test. Nonparametric tests were used in cases of non-normal distributions. Outliers (±3 *SD* from mean) in primary dependent variables were retained in the ﬁnal analyses when the results, including and excluding the outlier, were similar. All statistical analyses were performed using SPSS (IBM Corp., Chicago, IL, USA, version 28.0.1.0), and a two-sided *p*-value of 0.05 was used to determine statistical significance.

To assess differences between sexes at baseline, an independent t test was used for continuous normally distributed data, a Mann-Whitney U test was used for continuous nonparametric data, and a chi-square was used for categorical data. The change of continuous study parameters over time was assessed with either a paired samples T test or a Wilcoxon signed rank test.

Linear mixed models were used to test for associations between changes in protein intake with changes in bone markers (IGF-1, PTH, PINP, CTX) and QUS parameters. Three models of increasing complexity were built to adjust for confounding factors, which were based on the literature [40]–[44]. In the first model, only time was added as fixed factor. The second model was adjusted for age, sex, BMI, CFS, history of fractures, vitamin D status, and calcium supplementation. Additionally, the third model adjusted for the number of drugs, smoking, and alcohol. Models 1–3 were performed for males and females separately. The BMD was only measured at 3 months; therefore, time was not added to the model, and the association between the baseline variables and BMD at 3 months was tested. Since changes in BMD are more likely to be detected after at least 6 months [16],[41], it was assumed that BMD was stable from baseline till 3 months, which justified the investigation of the association between the baseline variables with BMD at 3 months.

The associations between nutritional status with QUS parameters and BMD were also tested using linear mixed models. These models were the same as described for protein; however, BMI was not included as a confounder because BMI is part of the MNA-SF.

## 3. Results

### 3.1. Study population

**Figure 1.**
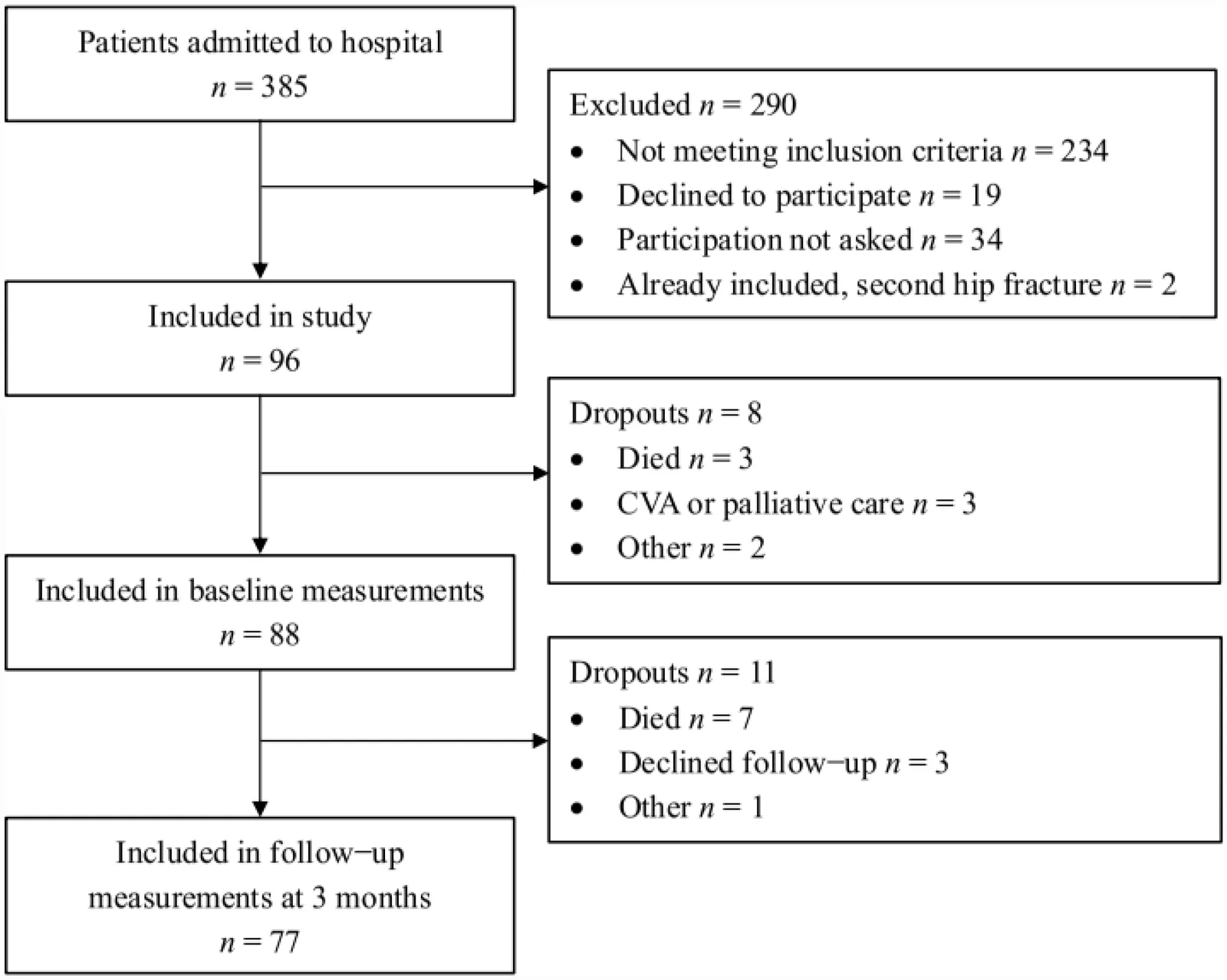
Flow diagram of participant selection.

Between September 2021 and September 2022, a total of 385 older hip fracture patients were admitted to the emergency department. A total of 96 were included in this study; baseline measurements were collected for 88 patients and follow-up measurements were collected for 77 patients (Figure 1). Physical measurements were not collected for all 88 patients at baseline due to either consent withdrawal (*n* = 3) or the inability to take measurements (*n* = 3). Likewise, at follow-up, either consent was withdrawn for physical measurements (*n* = 5) or the patients were not able or not willing to come to the hospital (*n* = 7). However, questionnaires could be completed using the records of patients who declined a follow-up but agreed to data retrieval, as well as for patients whose date of death was close to 3 months after hospital discharge and with their recent data available. In total, 12.6% of the patients died before the follow-up period at 3 months.

The patients had a mean age of 84 ± 6 years, with a median BMI of 27.2 kg/m^2^ (*IQR* 24.0–30.1), and 63% were female (Table 1). Statistically significant differences between males and females were found for weight, smoking habits, alcohol use, having a partner, CFS, previous fractures, osteoporosis diagnosis, and CRP levels. The median duration from hospital admission until the baseline measurements was 4 days, and 80% of the patients had surgery within 1 day after admission. Hemi-arthroplasty was the most common surgical method (55%), followed by TFN-Advanced Proximal Femoral Nailing System (TFNA, 28%), extended TFNA (13%), and other techniques (4%). The median length of required hospital stay was 6 days. A small part of the study population used a type of osteoporosis medication: 6% used corticosteroids, 3% used bisphosphonates, 1% used oestrogen or a similar hormone replacement, and 2% used some other osteoporosis medication. The cognitive assessment (MoCA) took place at the follow-up visit (*n* = 65); the median score was 25 (*IQR* 21–27), and 54% scored below the cut-off score of 26. At discharge, 5% went home without home care, 21% went home with home care, 68% went to a rehabilitation center, 1% went to a hospice, and 5% died during the hospital stay. The median length of stay in the rehabilitation centers was 35 days (*IQR*: 25–50). The Barthel Index significantly decreased from baseline (median 20, *IQR* 18–20) till discharge (median 13, *IQR* 11–15).

**Table 1.** Characteristics of the included hip fracture patients.

	*n*	All	Males	Females
Total participants, *n* (%)		95 (100)	35 (37)	60 (63)
Age, y, *mean* ± *SD*	95	84 ± 6	83 ± 7	84 ± 6
Weight, kg, *median* [*IQR*]	95	71.6 [62.5–80.0]	78.5 (71.3–85.3)*	69.7 (61.7–77.6)*
BMI, kg/m^2^, *median* [*IQR*]	95	27.2 [24.0–30.1]	26.1 (23.6–28.9)	27.5 (24.8–30.7)
Education level	86			
Primary		13 (15)	5 (15)	8 (15)
Lower secondary, *n* (%)		27 (31)	7 (21)	20 (38)
Upper secondary, *n* (%)		29 (34)	14 (41)	15 (29)
Higher, *n* (%)		17 (20)	8 (24)	9 (17)
Smoking	94			
Never, *n* (%)		46 (49)	13 (37)*	33 (56)*
Former, *n* (%)		31 (33)	18 (51)*	13 (22)*
Current, *n* (%)		17 (18)	4 (11)*	13 (22)*
Alcohol use	94			
Yes, *n* (%)		43 (46)	22 (63)*	21 (36)*
Amount (glasses/day), *median* [*IQR*]		0.0 [0.0–4.7]	1.5 [0.0–7.0]*	0.0 [0.0–3.0]*
CFS, score	95			
3 - Managing well, *n* (%)		48 (51)	25 (71)*	23 (38)*
4 - Vulnerable, *n* (%)		24 (25)	6 (17)*	18 (30)*
5 - Mildly frail, *n* (%)		20 (21)	3 (8)*	17 (28)*
6 - Moderately frail, *n* (%)		3 (3)	1 (3)	2 (3)
Living situation	95			
At home without home care, *n* (%)		81 (85)	31 (89)	50 (83)
At home with home care, *n* (%)		14 (15)	4 (11)	10 (17)
Having a partner, *n* (%)	95	39 (41)	21 (60)*	18 (30)*
Cause of fracture	95			
Accident inside, *n* (%)		58 (61)	19 (54)	39 (65)
Accident outside, *n* (%)		23 (24)	10 (29)	13 (22)
Fall after feeling unwell, *n* (%)		12 (13)	5 (14)	7 (12)
Other, *n* (%)		2 (2)	1 (3)	1 (2)
Fractures in the past, yes, *n* (%)	95	47 (49)	12 (34)*	35 (58)*
Hip fractures in the past, yes, *n* (%)	95	10 (11)	4 (11)	6 (10)
Vertebral fracture in the past, yes, *n* (%)	95	5 (5)	1 (3)	4 (7)
Comorbidities	95			
CCI, points, *median* [*IQR*]		1 [0–3]	2 [0–5]	1 [1–2]
Heart failure, *n* (%)		15 (16)	7 (20)	8 (13)
Peripheral vascular disease, *n* (%)		25 (26)	12 (34)	13 (22)
Pulmonary disorders, *n* (%)		14 (15)	6 (17)	8 (13)
Diabetes, *n* (%)		22 (23)	7 (20)	15 (25)
Kidney disease, *n* (%)		24 (25)	10 (29)	14 (23)
Cancer, *n* (%)		10 (11)	6 (17)	4 (7)
Osteoporosis, *n* (%)		15 (16)	1 (3)*	14 (23)*
Medication use at discharge	95			
Number of different drugs, *median* [*IQR*]		8 [6–12]	7 [5–13]	8 [7–11]
Polypharmacy^a^, *n* (%)		87 (92)	32 (91)	55 (92)
Vitamin D supplementation, *n* (%)		74 (78)	26 (74)	48 (80)
Calcium supplementation, *n* (%)		47 (49)	18 (51)	29 (48)
Length of hospital stay, days, *median* [*IQR*]	92	7.0 [5.5–9.0]	8.0 [6.0–11.0]	7.0 [5.0–9.0]
Length of necessary hospital stay, days^b^, *median* [*IQR*]	92	6.0 [5.0–8.0]	6.0 [5.0–9.0]	6.0 [5.0–8.0]
Complications during hospital stay	95			
Anemia, *n* (%)		23 (24)	8 (23)	15 (25)
Delirium, *n* (%)		11 (12)	4 (11)	7 (12)
Fever, *n* (%)		12 (13)	6 (17)	6 (10)
Hypotension, *n* (%)		16 (17)	4 (11)	12 (20)
Infections, *n* (%)		8 (9)	3 (9)	5 (8)
None, *n* (%)		38 (40)	16 (46)	22 (37)
Inflammation				
CRP, mg/L, *median* [*IQR*]	93	88 [50–128]	108 [64–155]*	74 [44–109]*
Leukocytes, 10^9^/L, *median* [*IQR*]	92	10.6 [9.1–12.5]	11.4 [8.3–12.7]	10.6 [9.3–12.1]
eGFR, ml/min/1,73m^2^, *median* [*IQR*]	87	66 [48–80]	65 [48–80]	67 [49–80]

Note: BMI = Body Mass Index, CCI = Charlson Comorbidity Index, CRP = C-reactive protein, eGFR = estimated glomerular filtration rate. ^a^ When using at least 5 drugs at the same time. ^b^ Days until hospital indication has ended, but who were forced to stay because there was not yet place in a rehabilitation center. * Significant difference between sexes (p-value < 0.05).

### 3.2. Protein intake and nutritional status

At baseline, the protein intake was <0.8 g/kg/d in 50% of the patients, between 0.8 and 1.2 in 43% of patients, and ≥1.2 g/kg/d in 7% of patients. The protein intake in grams remained stable over time (Table 2), while the protein intake in g/kg/d significantly increased (due to weight loss). The median weight change was −3.6 kg (*IQR*: −6.2 to −1.5; *p* < 0.001 from baseline). At baseline, 6% consumed protein-enriched products, which increased to 13% at 3 months; the protein content of these products was incorporated in the total protein intake. The patient's nutritional status worsened over time. The percentage of patients at risk of malnutrition increased from 19% to 51%, and those being malnourished increased from 1% to 13%.

### 3.3. Calcaneal quantitative ultrasound

A QUS could not be performed in all patients at baseline (*n* = 61), either due to an impossible transfer from bed to chair or a failure of the device. All three QUS parameters stayed similar over time (Table 2).

**Table 2.** Change in protein intake, nutritional status, QUS parameters and bone markers over time.

	*n*	Baseline	*n*	3 months	*P* value
*Protein intake and nutritional status*
Total protein intake, g/d, *mean ± SD*	86	58.4 ± 14.7	75	60.9 ± 15.7	0.14
Total protein intake, g/kg/d, *median* [*IQR*]	86	0.79 [0.64–0.97]	75	0.84 [0.72–1.05]	**0.001**
MNA-SF, score (0–14), *median* [*IQR*]	95	13 [12–14]	84	11 [9–13]	**0.001**
Malnourished, *n* (%)		1 (1)		11 (13)	**<0.001**
At risk of malnutrition, *n* (%)		18 (19)		43 (51)	
Normal nutritional status, *n* (%)		76 (80)		30 (36)	
*QUS parameters*
BUA, dB/MHz, *mean ± SD*	61	99.8 ± 11.6	60	101.7 ± 12.9	0.22
SOS, m/s, *mean ± SD*	61	1519.8 ± 32.3	60	1524.3 ± 34.2	0.51
SI, *mean ± SD*	61	72.0 ± 15.6	60	74.5 ± 17.0	0.63
*Bone markers*
Serum PINP, µg/L, *median* [*IQR*]	90	31.1 [20.3–42.6]	67	93.6 [65.4–119.4]	**<0.001**
Serum CTX, µg/L, *median* [*IQR*]	88	0.466 [0.303–0.759]	67	0.540 [0.330–0.763]	0.69
Serum IGF-1, nmol/L, *median* [*IQR*]	90	9.5 [7.6–12.4]	67	13.2 [10.6–18.4]	**<0.001**
Serum PTH, pmol/L, *median* [*IQR*]	93	7.8 [6.3–12.3]	68	5.2 [4.1–6.7]	**<0.001**
Serum 25(OH)D, nmol/L, *median* [*IQR*]	86	42 [28–56]	70	68 [53–80]	**<0.001**
25(OH)D <10 nmol/L, *n* (%)^a^	93	7 (8)	70	0 (0)	

Mote: BUA = broadband ultrasound attenuation, CTX = C-terminal telopeptide of type I collagen, IGF-1 = insulin like growth factor, MNA-SF = Mini Nutritional Assessment Short Form, PINP = procollagen type 1 N propeptide, PTH = parathyroid hormone, 25(OH)D = 25-hydroxyvitamin D, SI = Stiffness Index, SOS = speed of sound, QUS = quantitative ultrasound. ^a^ Patients below limit of detection.

### 3.4. Bone markers and vitamin D status

The median PINP levels increased from 31.1 µg/L (*IQR*: 20.3–42.6) at baseline to 93.6 µg/L (*IQR*: 65.4–119.4) at 3 months, while the CTX levels remained stable over time (Table 2). The IGF-1 levels increased from baseline (median: 9.5 nmol/L; *IQR*: 7.6–12.4) to 3 months (median: 13.2 nmol/L; *IQR*: 10.6–18.4). The PTH levels decreased over time and were above the reference levels (>9.3 pmol/L) in 42% of the patients at baseline, which declined to 8.8% of the patients at 3 months.

The serum 25(OH)D levels significantly increased over time. Patients with a deficiency (<25 nmol/L) changed from 26% at baseline to 3% at 3 months, and patients with an insufficiency (≥25 to <50 nmol/L) changed from 46% to 16%. Patients with adequate levels (≥50 nmol/L) increased from 28% to 81% and those with levels ≥70 nmol/L increased as well (from 8.6% to 46%).

### 3.5. DXA

Fifty-six patients underwent a DXA-scan at 3 months (Table 3). Lumber spine BMD was possibly overestimated in 30% of the patients due to scoliosis, sclerosis, or condensed structure after a compression fracture. One extreme outlier for L2–L4 BMD was removed for this reason. While only 16% of the patients was previously diagnosed with osteoporosis (extracted from patient record) at baseline, 34% of the patients were diagnosed with osteoporosis (*T*-score total hip ≤ −2.5 *SD*) and 52% of the patients had osteopenia (*T*-score total hip between −1 and −2.5 *SD*) at 3 months. These diagnoses at 3 months were not different between sexes.

**Table 3.** Bone mineral content and density of 56 patients at 3 months derived from DXA^a^.

Outcome	Values
Total body BMD, g/cm^2^	1.011 [0.870–1.076]
Total body BMC, g	2086 [1638–2533]
Spine BMD, g/cm^2^	1.092 [0.985–1.194]
L2L4 BMD, g/cm^2^	1.189 [1.017–1.298]
Total hip BMD, g/cm^2^	0.806 [0.658–0.876]
*T*-score total hip	−2.1 (0.9)
Femoral neck BMD, g/cm^2^	0.737 [0.667–0.792]
Ward's triangle BMD, g/cm^2^	0.481 [0.427–0.583]
Trochanter BMD, g/cm^2^	0.667 [0.549–0.727]
Pelvis BMD, g/cm^2^	0.795 [0.718–0.898]

Note: BMC = bone mineral content, BMD = bone mineral density, DXA = dual-energy X-ray absorption. ^a^ Values are median [*IQR*] or mean (*SD*).

### 3.6. Exploratory associations

No adjusted associations were found between the dietary protein intake and the IGF-1, PTH, PINP, CTX, QUS parameters, and BMD in the total sample (Supplementary Table S1). Sex-stratified fully adjusted models showed that there was an association between protein intake and the QUS parameter BUA (estimate 0.123, 95% *CI* 0.022–0.223) in females, but not in males. An additional analysis showed that higher IGF-1 levels was associated with higher PINP levels (fully adjusted model: estimate 1.215, 95% *CI* 0.363–2.066), but not with CTX levels (estimate 0.002, 95% *CI* −0.008–0.012). The PTH levels were not associated with the PINP and CTX levels.

In fully adjusted models, higher pre-fracture MNA-SF was associated with higher BMD in the total body (estimate 0.048, 95% *CI* 0.015–0.080), spine (estimate 0.085, 95% *CI* 0.025–0.144), total hip (estimate 0.055, 95% *CI* 0.018–0.093), and trochanter (estimate 0.057, 95% *CI* 0.018–0.096), but not with BMD at other sites or the QUS parameters (Table 4, Supplementary Table S2).

The QUS and DXA measurements could not be taken for all participants. The baseline protein intake, frailty, and nutritional status were similar between patients with and without QUS data. Patients with missing DXA data were more often at risk of malnutrition (33%) compared to those with DXA data (9%); however, the frailty scores were similar between the groups.

**Table 4.** Associations between MNA-SF with QUS and BMD in older hip fracture patients.

Exposure	Outcome	Model	Estimate	*SE*	95% *CI*	*P* value
	*QUS parameters^b^*					
ΔMNA-SF, score (0–14) ^a^	ΔBUA, dB/MHz	3	0.26	0.37	−0.48–1.00	0.48
	ΔSOS, m/s	3	−0.96	1.02	−3.00–1.09	0.35
	ΔSI	3	−0.29	0.41	−1.12–0.53	0.48
	*BMD^c^*					
Pre-fracture MNA-SF, score (0–14)	Total body BMD at 3 mo, g/cm^2^	3	0.048	0.016	0.015–0.080	**0.005**
	Spine BMD at 3 mo, g/cm^2^	3	0.085	0.029	0.025–0.144	**0.006**
	L2L4 BMD at 3 mo, g/cm^2^	3	0.035	0.029	−0.024–0.095	0.24
	Total hip BMD at 3 mo, g/cm^2^	3	0.055	0.018	0.018–0.093	**0.005**
	Femoral neck BMD at 3 mo, g/cm^2^	3	0.029	0.020	−0.013–0.070	0.17
	Ward's triangle BMD at 3 mo, g/cm^2^	3	0.028	0.016	−0.005–0.060	0.092
	Trochanter BMD at 3 mo, g/cm^2^	3	0.057	0.019	0.018–0.096	**0.006**
	Pelvis BMD at 3 mo, g/cm^2^	3	0.048	0.024	−0.0002–0.096	0.051

Note: BMD = bone mineral density, BUA = broadband ultrasound attenuation, MNA-SF = Mini Nutritional Assessment Short Form, SI = Stiffness Index, SOS = speed of sound, QUS = quantitative ultrasound. ^a^ Delta (Δ) refers to the change from baseline till 3 months. ^b^ Model 3: adjusted for time, age, sex, clinical frailty scale, history of fractures, vitamin D status, calcium supplementation, number of drugs, smoking, and alcohol. ^c^ Model 3: adjusted for pre-fracture age, sex, clinical frailty scale, history of fractures, vitamin D status, calcium supplementation, number of drugs, smoking, and alcohol.

## 4. Discussion

This study found no associations between protein intake and the bone markers, QUS parameters, and BMD in the total sample of older hip fracture patients; however, there was an association between protein intake and the QUS parameter BUA in females. A higher pre-fracture nutritional status was associated with higher total body, spine, total hip, and trochanter BMD. Regarding the bone markers, the PINP and IGF-1 levels increased from baseline to 3 months, while the CTX levels remained stable and the PTH levels decreased. The IGF-1 levels were associated with the PINP levels.

Contrary to our hypothesis, no associations were found between the dietary protein intake and the bone markers, QUS parameters, and BMD in the total sample. A possible explanation for this observation is that the results of the protein screener were affected by recall bias. In addition, this tool was not yet validated and, therefore, might produce inaccurate information. Nevertheless, similar intakes at baseline were found as a previous study in a similar study population which filled in food records through a combination of observations and weighing [8]. Previous studies that investigated the association between protein and bone health have found mixed results. Additionally, two studies in community-dwelling older adults found no differences in bone turnover markers between high *vs*. low protein intake after 3 years of follow-up [21],[22]. Regarding the QUS parameters, protein intake was solely associated with BUA in females for the current study. Previously, it was found that for Korean adults who consumed relatively low protein diets, meat protein intake was positively associated with the QUS parameter SI in older males, but not in older females [23]. The Korean adults were much younger than our study population (delta 24 years), consumed low protein diets, and had lower BMIs (delta 2 kg/m^2^); therefore, the studies are difficult to compare. Regarding the BMD, it was previously shown that total protein intake was associated with a higher total body and spine BMD in older adults [45]. However, these older adults were younger than the current study, had higher total protein intakes and better nutritional statuses, and consisted, for a large part, of healthy older adults. Recently, a prospective study that followed 26,318 women, aged 35–69 years, for a median time of 22.3 years found that a 25 g/d increment of protein was associated with a 14% reduced hip fracture risk [46]. Although there are several trials that are investigating the effect of oral administration of protein on nutritional, clinical or functional outcomes in hip fracture patients [47], a well-designed trial (e.g., sufficient sample size, administration duration, dose, adherence, longitudinal BMD measurements, and follow-up period) on a broad variation of outcomes is missing.

Although dietary intake, including protein intake, is one of the determinants of malnutrition, we solely found associations for nutritional status. Three components of the MNA-SF (weight loss, mobility, and BMI) are known to be associated with BMD. This may either reflect the limitations of the protein intake tool or suggest that the functional status (captured by MNA-SF) is a more proximal driver of bone health in this population than protein intake alone. This study found that a better pre-fracture nutritional status was associated with higher BMD at multiple sites. For every unit increase in MNA-SF, the predicted BMD was higher by 0.048 g/cm^2^ in the total body, 0.085 g/cm^2^ in the spine, 0.055 g/cm^2^ in the total hip, and 0.057 g/cm^2^ in the trochanter. Using the measured BMD values in this study, this reflects a higher BMD of 4.7%, 7.8%, 6.8%, and 8.5% in the total body, spine, total hip, and trochanter, respectively. Considering that the rate of bone loss is 0.5%–1% per year in older men and about 0.5%–2% in older women (depending on age since annual loss is faster in the first years after menopause) [48]–[51], this is a clinically relevant result. It should be acknowledged that the majority of the sample was classified as having a normal nutritional status, which raises the possibility that the observed association may be driven by a smaller subset of participants with both a low MNA-SF and a low BMD. Future studies with a greater variability in nutritional status could help clarify whether this association is robust across the full spectrum of MNA-SF scores.

According to the European Society of Parenteral and Enteral Nutrition (ESPEN) guidelines, older patients with a hip fracture should receive nutritional supplements to either maintain or improve their nutritional status [52], which is not always achieved in clinical practice (as seen in the current study). The need for nutritional support was also reflected in the data on protein intake and nutritional status. The protein intake was <0.8 g/kg/d in 50% of the patients and <1.2 g/kg/day in 93% of the patients, which is in line with previous studies [8]–[11]. The protein intake per g/kg/d significantly increased over time, which can be attributed to the median weight loss of −3.6 kg. This is an alarming finding, since weight loss is associated with bone loss and an increased fracture risk [48],[53]. Even though the consumption of protein-enriched products increased over time (from 6 to 13% of the patients), the total protein intake in grams remained stable. The combined prevalence of risk of malnutrition and malnourishment even increased from 20% at hospital admission to 64% at 3 months. This, again [8], emphasizes the importance of improved nutritional support in older hip fracture patients in order to increase their protein intake and to prevent weight loss and malnutrition.

Another aim of this study was to investigate how bone turnover markers change from post-surgery till 3 months. It was found that the PINP levels tripled over time, while the CTX levels remained stable. These findings are in line with the study by Stewart et al., which found that the PINP levels increased from baseline to 2 months in older hip fracture patients, while the CTX levels did not change [17]. However, the baseline measurement was performed at 2 weeks post-fracture, meaning that the levels were probably already elevated. Indeed, the baseline PINP levels were higher in the study of Stewart compared to the current study: mean 117.9 *vs*. median 31.1 µg/L, respectively. Likewise, the baseline CTX levels were higher (mean 0.91 *vs*. median 0.47 µg/L, respectively). Additionally, in Chinese hip fracture patients, the PINP levels tripled from baseline till 30–60 days, and was still elevated at 180–230 days. The CTX levels increased 1.5 times, was decreased by a large extent at 80–120 days, and was almost back at baseline after 180–230 days. Since the CTX levels were measured at 90 days in the current study, it could be that a peak for the CTX levels was missed. The increase in the PINP levels was probably caused by IGF-1, which is known to stimulate the proliferation and differentiation of osteoblasts [13]. The results of the current study were in line with this mechanism; higher IGF-1 levels were associated with higher PINP levels. Additionally, there was a decrease in the PTH levels, which may relate to a decreased bone resorption; however, no association with CTX (or PINP) was found.

Moreover, the serum 25(OH)D levels increased over time, which led to a remarkable decrease in patients with a deficiency or insufficiency. At 3 months, 81% of the patients had adequate levels (≥50 nmol/L), while this was only 28% at baseline. The reason for this improvement is that patients were prescribed vitamin D supplements after the laboratory results showed a vitamin D deficiency. Measuring the serum 25(OH)D levels is not standard care; however, based on these results, this might be implemented. Vitamin D supplementation of 800–1000 IU daily is recommended for this population [54],[55].

There were several limitations of this study. First, the tool to assess protein intake has not been validated. However, this tool was chosen since it is non-invasive and not time-consuming; thus, there is no burden for the patient, especially compared to the food frequency questionnaire and food records. Nevertheless, similar intakes at baseline were found to a previous study in a similar study population, which filled in food records through a combination of observations and weighing [8]. Second, the results are generalizable to a part of the older hip fracture patients, since patients with preexisting dementia were excluded and only community-dwelling older adults were included. However, patients with cognitive impairment were included, as reflected by the MoCA score at 3 months follow-up. Third, no adjustments were made to physical activity levels and energy intake. However, these variables are correlated to BMI and frailty, which were adjusted. In addition, instead of calcium intake, only an adjustment for calcium supplementation was possible. Lastly, the actual dropout was in line with the expected dropout (both 20%), but not all measurements could be performed at 3 months, which resulted in a higher dropout rate for some variables. However, this reflects the reality of everyday clinical practice in an older and fragile population. Additionally, the dropout rate had consequences for the power of the study. While the intention was to analyze models with a maximum of 8 variables for the associations with protein intake, model 3 was adjusted for 10 variables. However, multiple variables did not relevantly contribute to the model, and there were no large differences in the results between model 2 (adjusted for 7 variables) and 3. DXA-scans were not available for all participants; those with missing data were more often malnourished at baseline. This may have led to an underestimation of the true association between pre-fracture malnutrition and BMD.

## 5. Conclusions

To conclude, a good pre-fracture nutritional status was associated with a higher BMD in older hip fracture patients. The role of protein in supporting optimal bone health in these patients remains unclear, and well-designed trials with validated protein intake tools are needed. After a hip fracture, there is an increase in the PINP levels, which is probably caused by IGF-1, and the CTX levels remains stable over time. Strategies during rehabilitation seem warranted to prevent inadequate protein intakes, malnutrition, and weight loss.

## Use of AI tools declaration

The authors declare they have not used Artificial Intelligence (AI) tools in the creation of this article.


