# Supplementary material for: Protein intake, malnutrition, and its association with bone health after a hip fracture: A 3-month prospective study
Source: AIMS Public Health. 2025 Sep 26;12(3):970–88. doi: 10.3934/publichealth.2025048 (PMC12538243; doi:10.3934/publichealth.2025048)
Supplement: Supplementary file 1 [file publichealth-12-03-048-s001.pdf]

Public Health

AIMS Public Health, 12(3): 970–988.
DOI: 10.3934/publichealth.2025048
Received: 28 March 2025
Revised: 21 July 2025
Accepted: 05 August 2025
Published: 26 September 2025

https://www.aimspress.com/journal/aimsph

Research article

Protein intake, malnutrition, and its association with bone health after a hip fracture: A 3-month prospective study

Inge Groenendijk[1,*], Hugo H Wijnen[2], Diana G Taekema[2] and Lisette CPGM de Groot[1]

[1] Division of Human Nutrition and Health, Wageningen University & Research, Wageningen, The Netherlands
[2] Department of Geriatrics, Rijnstate Hospital, Arnhem, The Netherlands

* **Correspondence:** Email: inge.groenendijk@wur.nl.

Supplementary

Table S1. Explorative associations between protein intake and bone health outcomes in older hip fracture patients.

Exposure	Outcome	Model	Estimate	*SE*	95% *CI*	*P* value
	Bone markers[b]					
ΔProtein intake, g/d[a]	ΔIGF-1, nmol/L	1	0.025	0.027	−0.030–0.079	0.37
		2	0.018	0.030	−0.041–0.077	0.55
		3	0.019	0.030	−0.041–0.079	0.53
	ΔPTH, pmol/L	1	−0.013	0.018	−0.048–0.023	0.48
		2	−0.016	0.020	−0.057–0.024	0.43
		3	−0.013	0.020	−0.052–0.027	0.53
	ΔPINP, μg/L	1	0.037	0.15	−0.26–0.33	0.80
		2	0.21	0.15	−0.094–0.51	0.17
		3	0.17	0.16	−0.15–0.49	0.29
	ΔCTX, μg/L	1	−0.001	0.002	−0.005–0.002	0.47
		2	−0.001	0.002	−0.005–0.003	0.63
		3	−0.001	0.002	−0.005–0.002	0.46
	QUS parameters[b]					
ΔProtein intake, g/d	ΔBUA, dB/MHz	1	0.099	0.054	−0.009–0.21	0.072

		2	0.064	0.052	−0.040–0.17	0.22
		3	0.044	0.053	−0.061–0.15	0.40
	ΔSOS, m/s	1	−0.056	0.14	−0.34–0.23	0.70
		2	−0.075	0.16	−0.40–0.25	0.64
		3	−0.092	0.16	−0.40–0.22	0.56
	ΔSI	1	0.056	0.056	−0.055–0.17	0.32
		2	0.028	0.066	−0.10–0.16	0.68
		3	0.017	0.062	−0.11–0.14	0.78
	BMD[c]					
Pre-fracture protein intake, g/d	Total body BMD at 3 mo, g/cm^2	1	0.001	0.001	−0.002–0.003	0.58
		2	0.0004	0.001	0.001–0.002	0.64
		3	−0.0003	0.001	−0.003–0.002	0.79
	Spine BMD at 3 mo, g/cm^2	1	0.002	0.002	−0.002–0.005	0.32
		2	0.002	0.002	−0.002–0.005	0.27
		3	0.003	0.002	−0.002–0.007	0.19
	L2L4 BMD at 3 mo, g/cm^2	1	0.003	0.002	−0.006–0.001	0.15
		2	−0.002	0.002	−0.006–0.002	0.36
		3	−0.0004	0.002	−0.005–0.004	0.87
	Total hip BMD at 3 mo, g/cm^2	1	0.001	0.001	−0.001–0.003	0.43
		2	0.001	0.001	−0.001–0.003	0.29
		3	0.002	0.001	−0.001–0.005	0.11
	Femoral neck BMD at 3 mo, g/cm^2	1	0.002	0.001	0.0002–0.004	**0.036**
		2	0.002	0.001	−0.001–0.004	0.16
		3	0.003	0.002	−0.0005–0.006	0.092
	Ward's triangle BMD at 3 mo, g/cm^2	1	0.002	0.001	−0.0002–0.004	0.078
		2	0.001	0.001	−0.001–0.003	0.26
		3	0.002	0.001	−0.001–0.004	0.18
	Trochanter BMD at 3 mo, g/cm^2	1	0.001	0.001	−0.002–0.003	0.53
		2	0.001	0.001	−0.001–0.003	0.30
		3	0.003	0.001	−0.0003–0.006	0.076
	Pelvis BMD at 3 mo, g/cm^2	1	−0.0003	0.001	−0.003–0.002	0.83
		2	0.0002	0.001	−0.003–0.003	0.91
		3	−0.002	0.002	−0.005–0.002	0.34

Note: BMD = bone mineral density, BUA = broadband ultrasound attenuation, CTX = C-terminal telopeptide of type I collagen, IGF-1 = insulin like growth factor, PINP = procollagen type 1 N propeptide, SI = Stiffness Index, SOS = speed of sound, QUS = quantitative ultrasound. [a] Delta (Δ) refers to the change from baseline till 3 months. [b] Model 1: adjusted for time. Model 2: additionally adjusted for age, sex, BMI, CFS, history of fractures, vitamin D status, and calcium supplementation. Model 3: additionally adjusted for number of drugs, smoking, and alcohol. [c] Model 1: crude. Model 2: adjusted for pre-fracture age, sex, BMI, CFS, history of fractures, vitamin D status, and calcium supplementation. Model 3: additionally adjusted for pre-fracture number of drugs, smoking, and alcohol.

Table S2. Explorative associations between MNA-SF with QUS and BMD in older hip fracture patients.

Exposure	Outcome	Model	Estimate	*SE*	95% *CI*	*P* value
	QUS parameters[b]					
ΔMNA-SF, score (0–14)[a]	ΔBUA, dB/MHz	1	0.33	0.34	−0.35–1.01	0.34
		2	0.45	0.37	−0.28–1.18	0.22
		3	0.26	0.37	−0.48–1.00	0.48
	ΔSOS, m/s	1	−0.48	0.87	−2.22–1.25	0.58

			2	−0.25	1.05	−2.34–1.84	0.82
			3	−0.96	1.02	−3.00–1.09	0.35
		ΔSI	1	−0.064	0.33	−0.73–0.60	0.85
			2	0.17	0.44	−0.71–1.04	0.70
			3	−0.29	0.41	−1.12–0.53	0.48
		BMD[c]					
Pre-fracture score (0–14)	MNA-SF,	Total body BMD at 3 mo, g/cm^2	1	0.001	0.013	−0.25–0.027	0.94
			2	0.031	0.014	0.002–0.060	**0.035**
			3	0.048	0.016	0.015–0.080	**0.005**
		Spine BMD at 3 mo, g/cm^2	1	0.022	0.019	−0.016–0.059	0.25
			2	0.056	0.025	0.006–0.107	**0.029**
			3	0.085	0.029	0.025–0.144	**0.006**
		L2L4 BMD at 3 mo, g/cm^2	1	−0.017	0.019	−0.055–0.021	0.38
			2	0.004	0.026	−0.048–0.057	0.87
			3	0.035	0.029	−0.024–0.095	0.24
		Total hip BMD at 3 mo, g/cm^2	1	0.004	0.015	−0.025–0.034	0.77
			2	0.044	0.019	0.006–0.082	**0.025**
			3	0.055	0.018	0.018–0.093	**0.005**
		Femoral neck BMD at 3 mo, g/cm^2	1	−0.005	0.014	−0.032–0.023	0.73
			2	0.023	0.018	−0.014–0.060	0.22
			3	0.029	0.020	−0.013–0.070	0.17
		Ward's triangle BMD at 3 mo, g/cm^2	1	−0.003	0.012	−0.027–0.021	0.79
			2	0.022	0.016	−0.009–0.054	0.16
			3	0.028	0.016	−0.005–0.060	0.092
		Trochanter BMD at 3 mo, g/cm^2	1	0.017	0.015	−0.013–0.047	0.26
			2	0.044	0.019	0.005–0.082	**0.029**
			3	0.057	0.019	0.018–0.096	**0.006**
		Pelvis BMD at 3 mo, g/cm^2	1	0.001	0.015	−0.029–0.031	0.92
			2	0.026	0.021	−0.017–0.069	0.22
			3	0.048	0.024	−0.0002–0.096	0.051

Note: BMD = bone mineral density, BUA = broadband ultrasound attenuation, MNA-SF = Mini Nutritional Assessment Short Form, SI = Stiffness Index, SOS = speed of sound, QUS = quantitative ultrasound. [a] Delta (Δ) refers to the change from baseline till 3 months. [b] Model 1: adjusted for time. Model 2: additionally adjusted for age, sex, CFS, history of fractures, vitamin D status, and calcium supplementation. Model 3: additionally adjusted for number of drugs, smoking, and alcohol. [c] Model 1: crude. Model 2: adjusted for pre-fracture age, sex, CFS, history of fractures, vitamin D status, and calcium supplementation. Model 3: additionally adjusted for pre-fracture number of drugs, smoking, and alcohol.

AIMS Press

© 2025 the Author(s), licensee AIMS Press. This is an open access article distributed under the terms of the Creative Commons Attribution License (https://creativecommons.org/licenses/by/4.0)
